# Annotations

**Published:** 1904-01-23

**Authors:** 


### Annotations.

### The Teeth in Inherited Syphilis.

In the current number of *The Ophthalmoscope* Dr. Darier describes a condition of the first permanent molar teeth which he regards as analogous to the changes in the incisors and canines first noted by Mr. Hutchinson and equally with these significant of a syphilitic inheritance. He has observed the molars to be involved when the incisors and canines show no abnormality and hence is inclined to attach considerable diagnostic value to his observations. Dr. Darier's suggestion is that the particular dental changes in each individual case depend on the severity of the pathological process and the time at which this reaches its acme. Hence it is possible for the classical teeth to escape whilst distinct alterations occur in the first molars. The essential feature of these changes is an arrest of development coming into operation just before the summit of the tooth receives its covering of protective enamel. Consequently, at the time of eruption the tooth presents its four tubercles destitute of enamel and exposing the yellow dentine, whilst round the base of the tubercles is seen a "collar" corresponding to the line at which the enamel formation terminates. These appearances, it is obvious, are not likely to remain long unchanged, as they depend upon conditions which favour caries of the teeth, and, therefore, after a time nothing is to be seen but the crown of the tooth with a carious centre. It is only at, or shortly after, eruption of the tooth that the facts regarded by Dr. Darier as characteristic can be observed. The practical value of his observation is still further qualified by the fact that abnormal conditions of the first permanent molars may arise from the administration of mercury in infancy. Indeed, according to Mr. Hutchinson, they are the teeth which "especially mark the effects of mercury." It is obvious, therefore, that the facts Dr. Darier describes when existing apart from other evidence afford a somewhat uncertain ground on which to rest a diagnosis of inherited syphilis.

### Classics as School Books.

By a curious coincidence we lately read in two journals, of quite different nature, articles by two different writers who agree in lamenting the employment in the schoolroom of those poems and stories which we have agreed to regard as classic. Indeed an instance is given of a girl who avowed her hatred of Shakespeare and all his works because of what she had suffered from much enforced study of one play. Truly schoolmasters and the writers and publishers of educational books are people to be pitied. They cannot please their clients. If school books are dull or silly there is a complaint that, instead of cultivating the youthful mind by introducing it early to the masterpieces of literature, those who are responsible are wasting golden opportunities of elevating the rising generation. If they employ editions without notes, the protest is that there are so many allusions, so many old-world phrases that the children cannot understand, that no wonder the poor little souls don't like the books. And if they use notes, and ask the pupils to read them, they are accused of spoiling their future enjoyment of these very classics. But though boys and girls in their 'teens may profess to hate a book because it has been used in school, it does not follow that the distaste is permanent. Certain Greek and Roman classics have been used as text-books for many generations, yet still men find beauty in Homer and Horace. As for the much condemned notes, it is worth remembering that some writers—Scott, for example—did not wait for the educational editors, but provided notes of their own. And, having known a twelve year-old who frankly said he "didn't care for books" supplement a history lesson by a study of the notes to "Kenilworth," we are inclined to see a certain value even in these. The truth is that every age has its affectations, and that of the schoolboy and his sister is a scorn for all connected with school. But this will pass away, and in nine cases out of ten they will not love the classic authors less for having been introduced to them at the school which they now malign, but which they will one day look back to with affection.

### Trees and Climate.

There is some reason to believe that the recent troubles in Trinidad had their origin in the first instance in the decrease of the supply of rain-water caused by the denudation of the forests. The reckless destruction of timber has been largely the work of negro squatters who make clearings on the hill sides by setting fire to the trees, a common practice in Jamaica and other West India Islands, as well as Trinidad, has been going on for years with marked results for the worse in the matter of climate, as has been the case in Palestine, Cyprus, and other countries. But apart from the deterioration of climate there is the deplorable waste of valuable timber to be considered. In the United States they have long since awakened to the value of their once-despised woodland wealth and the unsuspected services it rendered, for on Planting Day the youngsters bed their saplings towards making up for Uncle Sam's past prodigality. In this country also we are becoming dimly conscious of the beneficial functions performed by trees, while in Ireland another grievance has been discovered in the disappearance of the vast growth of oak which once covered the island, and an agitation is now on foot to obtain official support and encouragement for tree planting on a large scale, such as that perhaps which we read of in the "Gentleman's Magazine" for 1783, wherein mention is made of a north country squire being handsomely rewarded by the Crown for having planted some 60,000 larch trees. Apart from the general utility of tree planting, the beautifying effects should not be forgotten. The suggested re-afforestation of the Black Country would do much to redeem the arid ugliness of many shale-covered Midland wastes, and the resuscitation of Hainault Forest (on what is now ploughed land) would add another sylvan charm to the outskirts of Greater London. The former scheme was proposed by Professor Fisher of the Royal Engineering College, Cooper's Hill, in 1894, when he estimated that 14,000 acres might be planted at a cost of £10 per acre, including fencing, and this, if pine, spruce, and larch were planted, would in 30 years yield pit timber worth 30s. the ton. Such afforestation has already been successful in the Forest of Dean and in France and Belgium on a similar soil of decomposing shale.

